# BosR Functions as a Repressor of the *ospAB* Operon in *Borrelia burgdorferi*

**DOI:** 10.1371/journal.pone.0109307

**Published:** 2014-10-01

**Authors:** Yanlin Shi, Poonam Dadhwal, Xin Li, Fang Ting Liang

**Affiliations:** 1 Department of Pathobiological Sciences, Louisiana State University, Baton Rouge, Louisiana, United States of America; 2 Department of Veterinary Biosciences, The Ohio State University, Columbus, Ohio, United States of America; The University of Texas at San Antonio, United States of America

## Abstract

The Lyme disease spirochete, *Borrelia burgdorferi*, must abundantly produce outer surface lipoprotein A (OspA) in the tick vector but downregulate OspA in mammals in order to evade the immune system and maintain its natural enzootic cycle. Here, we show that BosR binds two regulatory elements of the *ospAB* operon and that increasing BosR expression leads to downregulation of OspA. Both regulatory sequences, *cisI* and *cisII*, showed strong BosR-binding and *cisII* bound much tighter than *cisI*. A promoterless *bosR* gene fused with an inducible promoter was introduced into an *rpoS* mutant and a wild-type strain to assess RpoS-independent and -dependent downregulation of OspA by BosR. With the induction of BosR expression, OspA expression was reduced more significantly in the RpoS-deficient than wild-type background, but not completely repressed. In the presence of constitutive expression of OspC, DbpA and DbpB, increasing BosR production resulted in complete repression of OspA in the RpoS mutant. Taken together, the study clearly demonstrated BosR serves as a repressor that binds both regulatory elements of the *ospAB* operon and shuts off expression.

## Introduction

Outer surface proteins (Osps) A and B, encoded by a 2-gene operon [1], are among the most abundantly produced outer surface antigens by the Lyme disease spirochete, *Borrelia burgdorferi*, in engorged and unfed *Ixodes* ticks [2]–[5]. In response to a fresh bloodmeal, *B. burgdorferi* downregulates OspA/B and upregulates OspC and other proteins, a process that prepares *B. burgdorferi* for infection of a mammal [6]–[8]. Repressing *ospAB* expression during mammalian infection is critical for *B. burgdorferi* to evade the immune system, cause persistent infection, and maintain the enzootic cycle, as both OspA and OspB, even expressed at a low level, may ultimately induce a strong humoral response due to their high immunogenicity. The specific response can pose tremendous pressure on the pathogen or even clear infection [9], [10]. Even by chance, the anti-OspA/B humoral response may not effectively target spirochetes with very low OspA/B expression in mammalian tissues. Once acquired by the tick vector, the pathogen has to dramatically upregulate OspA/B and consequently becomes extremely vulnerable to the specific antibodies in bloodmeal [11], whereby potentially leading to the eradication of the organism and a discontinuation of the enzootic cycle.

Expression of the *ospAB* operon is driven by a *σ* ^70^-dependent promoter [12]. *B. burgdorferi* has only two alternative *σ* factors, RpoN and RpoS, which form a regulatory network, in which RpoS expression depends on RpoN and controls expression of many important Osps, including OspC, DbpA and DbpB [13]. A study by Radolf and colleagues suggested that RpoS is involved in repression of OspA expression [14]. Given the fact that RpoS activates expression of many Osps, the indirect effect on OspA expression due to their absence could be significant. The inability of *rpoS* mutants to downregulate OspA may be caused by an indirect effect resulting from the lack of RpoS-dependent Osps. There has been no evidence showing any interaction of RpoS with DNA sequences associated the *ospAB* operon, essentially ruling out direct involvement of RpoS in OspA downregulation.

Successful identification of two regulatory sequences, namely *cisI* and *cisII*, which flank the *ospAB* promoter, indicates the existence of a repressor(s), which should bind the two elements and shut off expression during murine infection [15]. Our recent study revealed that more than 156 genes in the *B. burgdorferi* genome have at least one putative BosR-binding site, among which is the *ospAB* locus [16]. Interestingly, one of the two putative BosR-binding sites associated with the *ospAB* operon is completely included within the previously identified *cisII* regulatory sequence [15], and the second partially overlaps with *cisII* and the -10 region of the promoter [16].

As a key regulator, BosR functions to bind the *rpoS* promoter region and positively regulate the alternative *σ* factor, which in turn upregulates a number of Osps, including OspC, DbpA and DbpB [17]–[19]. Although *in vitro* grown *B. burgdorferi* does not produce BosR during early growth phase, once grown to late log phase, the pathogen dramatically upregulates the regulator. This dramatic BosR upregulation thus far has not been correlated with OspA downregulation, seriously challenging the notion that BosR, in addition to the identified function, may also serve as a repressor of the *ospAB* operon. The current study first demonstrated that BosR bound both *cisI* and *cisII* and then showed that increased BosR expression indeed led to a shutoff of OspA.

## Materials and Methods

### Expression and purification of recombinant BosR

The entire *bosR*-coding region was amplified from genomic DNA of *B. burgdorferi* B31 with the use of primers P5F and P5R (Table 1). The resultant PCR product was digested, purified and cloned into pET-23a vector (EMD Chemicals Inc., Darmstadt, Germany), generating a construct that contained the *bosR*-coding region fused with a C-terminal His_6_ tag. One Shot BL21(DE3)pLysS Chemically Competent *E. coli* cells (Life Technologies, Grand Island, NY) were transformed with the construct and induced with 1.0 mM isopropyl-beta-D-thiogalactopyranoside (IPTG) (Sigma Chemical Co., St. Louis, MO). Recombinant BosR was affinity-purified with the use of HiTrap Chelating HP following the manufacturer’s instruction (GE Healthcare Bio-Sciences, Pittsburgh, PA). Protein concentration was measured using Quick Start Bradford Dye Reagent following the manufacturer’s protocol (Bio-Rad Laboratories, Hercules, CA).

**Table 1:** Primers used in the study^a^.

Primer	Sequence (5′ to 3′)
P1F	AAATTCATGCCATGGACGACAACATAATAGACG
P1R	TTTCCGCTCGAGTCATAAAGTGATTTCCTTGTTCTC
P2F	AAATCATGAACGACAACATAATAGACGTACATTCC
P2R	AAAGGATCCACCAGTATTAAGAGTAATAAGAATATAAG
P3F	AAAGCTAGCAGGAAACAGCTATGACCATGATTAC
P3R	TGCCAAGCTTGCATGCCTGCAG
P4F	GACCTGCAGGCATGCAAGCTTGG
P4R	AAAGCTAGCGTCTTGATTATCGGGCGAAGAG
P5F	TAATTCCATATGAACGACAACATAATAGACG
P5R	TTTCCGCTCGAGTAAAGTGATTTCCTTGTTCTC

a The underlined sequences are restriction enzyme sites: a BamHI site (P2R), a BspHI site (P2F), a NdeI site (P5F), a NcoI site (P1F), NheI sites (P3F and P4R), PstI sites (P3R and P4F), and XhoI sites (P1R and P5R).

### Gel mobility shift assay

Each strand of the probes *CisI, CisII* and IRs was synthesized by Integrated DNA Technologies, Inc. (Coralville, IA). One strand of each complementary pair was incorporated with 5′ digoxigenin during synthesis. Complementary strands were allowed to anneal to form a double-strand probe. The binding assay reaction volume was set in 20 µl, in which 1.0 nM DNA probe was allowed to bind 400 nM of recombinant BosR at room temperature for 10 min. The binding buffer contained 50 µg/ml salmon sperm DNA, 100 µg/ml BSA, 1.0 mM DTT, 50 mM KCl, 10 mM Tris (pH 7.5) and 5% glycerol. In the competition assay, 10 nM DNA competitor was first added to a reaction volume of 20 µl and allowed to interact with 400 nM of recombinant BosR at room temperature for 10 min before 1.0 nM DNA probe was supplemented and incubated for an additional 10 min. Resultant mixtures were separated by electrophoresis on 10% polyacrylamide gels prepared with 0.5x TBE buffer. Separated DNA was transferred to a nylon membrane and probed with the use of the DIG High Prime DNA Labeling and Detection Starter Kit I per the manufacturer’s instructions (Roche Applied Science, Mannheim, Germany).

### Construction of pIBM-*rpoS_in_*, pME22-*bosR’* and pME22-*C’B’A’*-*bosR’*

**Figure 1:**
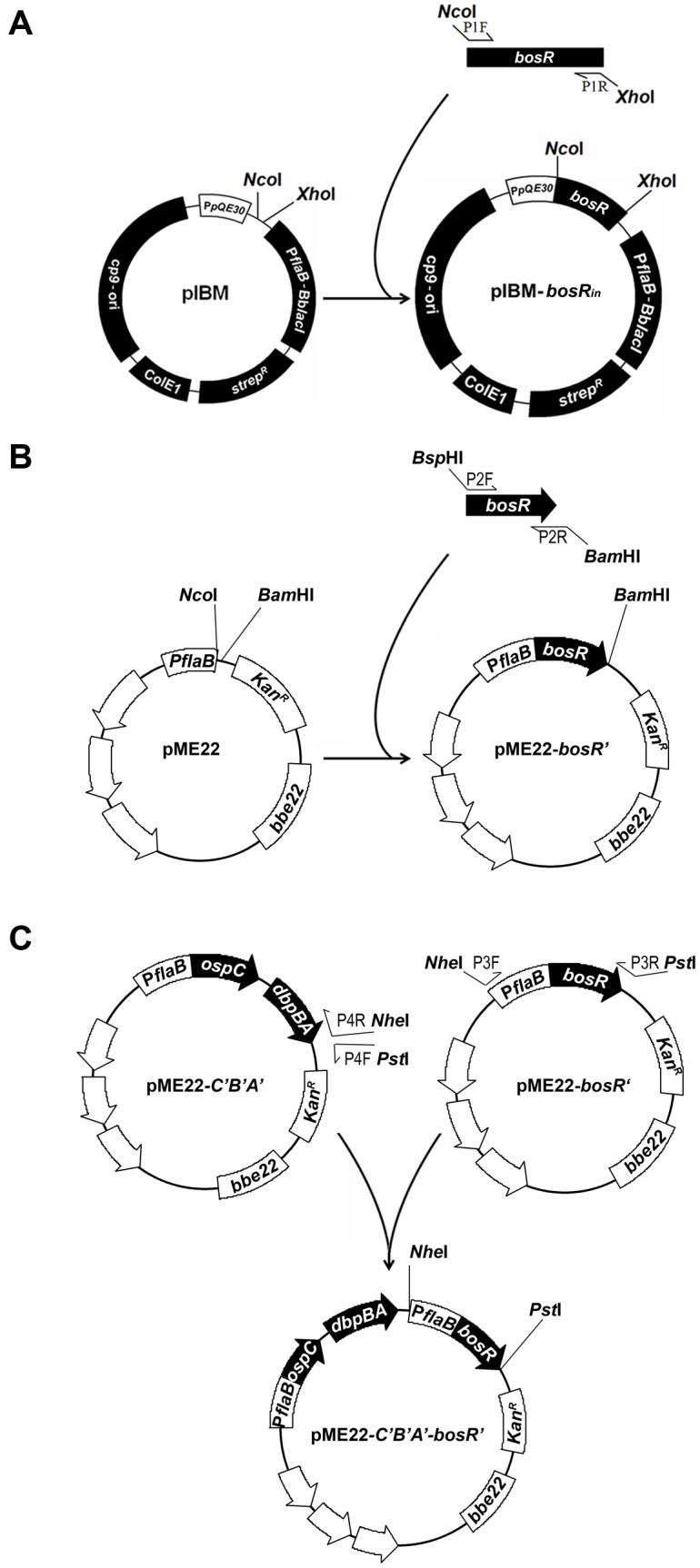
Construction of pIBM-*bosR_in_*, pME22-*bosR’* and pME22-*C’B’A’*-*bosR’*. (A) To construct pIBM-*bosR_in_*, a 554-bp DNA fragment covering the entire coding region of the *bosR* gene was amplified. The resultant PCR product was cloned into pIBM. (B) To construct pME22-*bosR’*, a 597-bp DNA fragment containing the entire coding sequence of the *bosR* gene was amplified and cloned into pME22. (C) To construct pME22-*C’B’A*-*bosR’*, a 917-bp DNA fragment covering the promoterless *bosR* gene fused with the *flaB* promoter was amplified from pME22-*bosR’*. A 10,059-bp DNA fragment harboring the promoterless *ospC*, *dbpB* and *dbpA* fused with the *flaB* promoter was amplified from pME22-*C’B’A’*. The resultant PCR products were ligated to complete construction of pME22-*C’B’A’*-*bosR’*.

As diagrammed in Fig. 1A, to construct pIBM-*bosR_in_*, a 554-bp DNA fragment covering the entire coding region of the *bosR* gene was amplified with the use of primers P1F and P1R (Table 1) and DNA template extracted from *B. burgdorferi* B31. The resultant PCR product was purified, digested with NcoI and XhoI, repurified and cloned into pIBM, which was created in a previous study [20], and pre-digested with the same enzymes.

To construct pME22-*bosR’*, the plasmid that conferred constitutive BosR expression, a 597-bp DNA fragment containing the entire coding sequence of the *bosR* gene was amplified with the use of primers P2F and P2R (Table 1) and DNA template purified from *B. burgdorferi* B31. The resultant amplicon was purified, digested with BspHI and BamHI, repurified and cloned into pME22, which was created in an earlier study [21], and pre-digested with NcoI and BamHI. The construction process was summarized in Fig. 1B.

To construct pME22-*C’B’A’*-*bosR’*, a plasmid that was able to provide constitutive BosR, OspC, DbpA and DbpB expression simultaneously, two plasmids, pME22-*bosR’* and pME22-*C’B’A’* were used. pME22-*C’B’A’* was constructed in an earlier study [20]. A 917-bp DNA fragment covering the promoterless *bosR* gene fused with the *flaB* promoter was amplified from pME22-*bosR* with use of primers P3F and P3R (Fig. 1C; Table 1). A 10,059-bp DNA fragment harboring the promoterless *ospC*, *dbpB* and *dbpA* fused with the same *flaB* promoter was amplified from pME22-*C’B’A’* by using primers P4F and P4R (Fig. 1C; Table 1). The resultant PCR products were pooled, purified, digested with NheI and PstI, and ligated to complete construction of pME22-*C’B’A’*-*bosR’*.

### Generation of transformants

The *rpoS* mutant, Δ*rpoS*, which was generated in our previous study [20], was grown to late logarithmic (log) phase in Barbour-Stoenner-Kelly H (BSK-H) complete medium (Sigma). Spirochetes were harvested from approximately 40 ml of culture and transformed with pIBM-*bosR_in_*, pME22-*bosR’* or pME22-*C’B’A’*-*bosR’* as described previously [22]. Transformants were identified by PCR using a primer pair specific for either streptomycin or kanamycin cassette and their plasmid content was analyzed as described previously [22].

### Growth rate estimation

The spirochete culture was grown at 33°C to late log phase (approximately 10^8^ cells/ml) in BSK-H complete medium and diluted to 10^5^ cells/ml with the medium. A total of fifteen 1.3-ml aliquots were prepared and IPTG was then added at final concentrations of 0, 0.02, 0.05, 0.10 and 0.20 mM. Each inducer concentration was in triplicate. All aliquots were incubated at 33°C and cell numbers were counted daily for 10 days. Either the parental clone 13A or the transformant Δ*rpoS*/*rpoS* was used as a control.

### Immunoblotting and Coomassie staining

Spirochetes were harvested by centrifugation at 5,000×*g* for 10 min at 4°C. Resultant pellets were dissolved in a SDS-PAGE sample buffer, separated by electrophoresis and electrotransferred onto nitrocellulose membranes. Blots were probed either with a mixture of FlaB mAb and mouse anti-BosR sera or OspC mAb, mouse anti-DbpA or anti-DbpB sera alone as described in our previous study [23]. Mouse BosR sera was prepared in an earlier study [16]. For protein analysis, proteins separated on SDS-PAGE gels were directly stained with Coomassie Brilliant Blue G-250 (Amresco, Inc., Solon, OH).

### qRT-PCR

Total RNA was prepared from cultured spirochetes, converted to cDNA through reverse-transcription and quantified for the mRNA copy numbers of *flaB* and *ospA* by quantitative PCR as described previously [24].

## Results

### Both *ospA* regulatory sequences, *cisI* and *cisII*, bind BosR, albeit *cisII* shows much stronger binding

**Figure 2:**
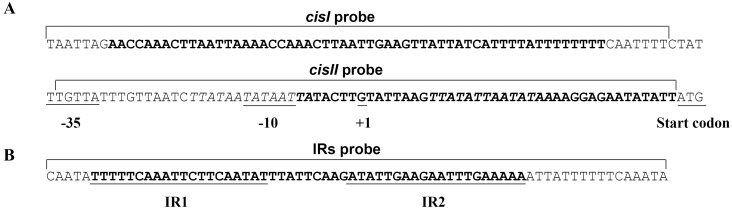
Diagram of *cisI*, *cisII* and putative BosR-binding sites of the *ospAB* operon and three probe sequences used in the study. (A) The location of *cisI*, *cisII* and putative BosR-binding sites and the sequences of *cisI* and *cisII* probes. The regulatory sequences upstream of the coding region of the *ospAB* operon include *cisI* and *cisII* (both in bold) and two putative BosR-binding sites (italic). One putative site overlaps with the -10 region and *cisII*, and the other is contained within *cisII*. The -35 and -10 regions of the promoter, transcriptional initiation site, and start codon ATG all are underlined. The sequences of *cisI* and *cisII* probes are marked with brackets. (B) The IRs were identified as the *ospC* operator in a previous study [25], and used as a control probe. The sequence of the probe is marked with a bracket.

Our previous study identified two regulatory sequences contributing to downregulation of *ospA* expression in the mammalian host located upstream of the *ospAB* promoter and located between the promoter and the translational start codon, namely *cisI* and *cisII*, respectively [15]. Our most recent study revealed the existence of two putative BosR-binding sites associated with the *ospAB* locus [16]. One of the sites is completely included within *cisII* and the second covers the entire -10 region of the *ospAB* promoter and the first two base pairs of *cisII* (Fig. 2A). As the second site of the 14-bp putative BosR-binding sequence overlaps with the -10 region, it is impossible to entirely remove it without inactivation of the *ospAB* promoter. For this particular reason, only 2 bps of this putative binding site were identified as a part of *cisII* in the previous study [15].

**Figure 3:**
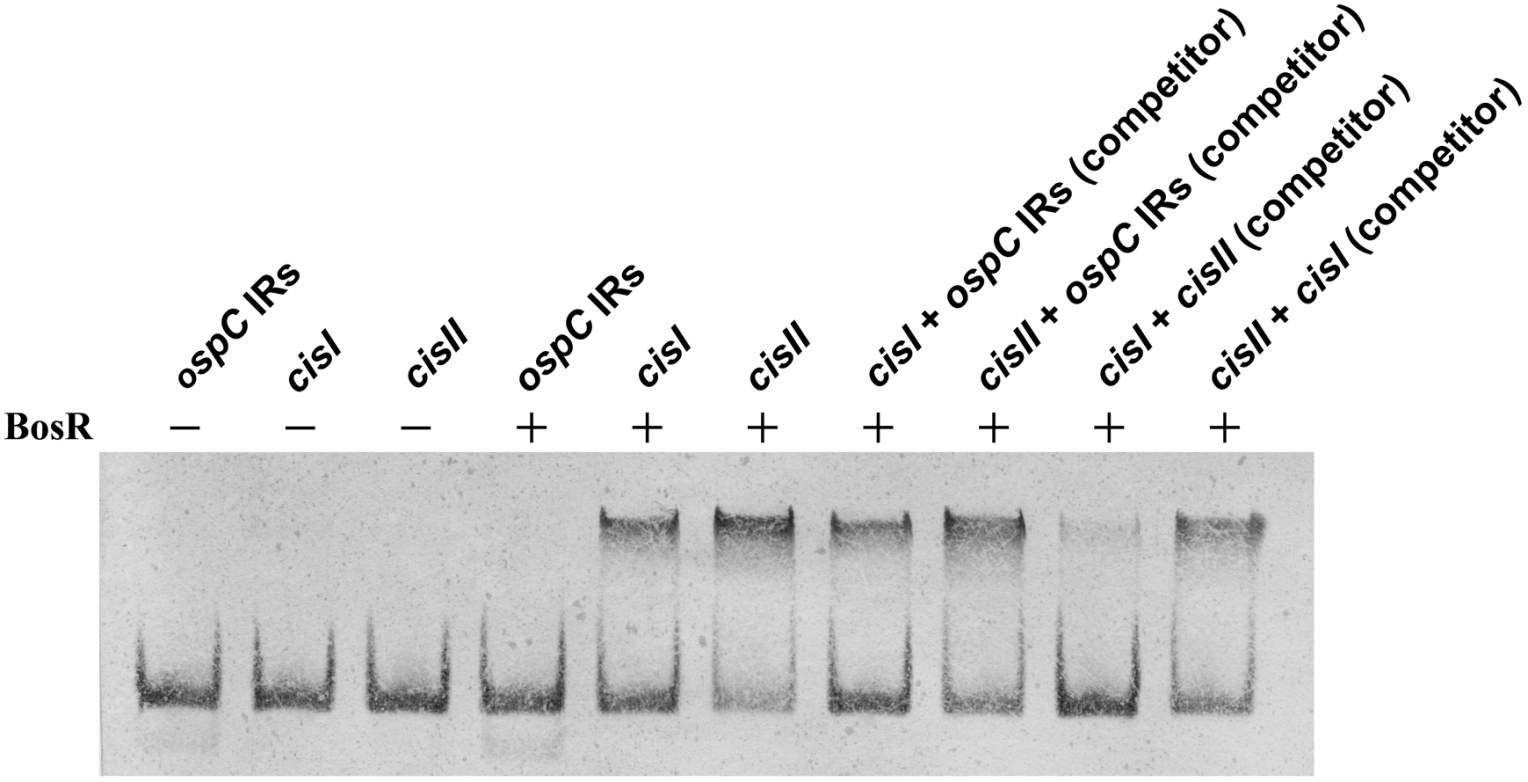
Both regulatory sequences of the *ospAB* operon, *cisI* and *cisII*, bind BosR and *cisII* shows stronger binding. The binding buffer contained 50 µg/ml salmon sperm DNA. The ratio of a DNA probe and a competitor was set at 1∶10 (1.0/10 nM). Mobility shift analysis was performed with 10% polyacrylamide gels.

The regulatory sequence *cisI* contains no putative BosR-binding site but showed a critical role in repression of *ospA* transcription during murine infection, albeit it is less effective than *cisII* in this regard [15]. To examine if the previously identified regulatory elements were able to bind BosR, three 70-bp DNA probes, namely *cisI*, *cisII* and IRs, were designed and synthesized as diagrammed in Fig. 2. Probe *cisI* covered the entire *cisI* sequence and additional 14 bps up- and downstream sequences. Probe *cisII* included the entire *cisII* sequence and extended upstream to include the -35 region of the *ospAB* promoter. Probe IRs contained the *ospC* IRs and extended few bps up- and downstream to make up a total of 70 bps. The IRs sequence was previously identified as an operator of the *ospC* gene and was not expected to bind BosR; thus, it was chosen as a negative control [25]. To minimize nonspecific DNA interaction with BosR, the binding buffer was supplemented with 50 µg/ml salmon sperm DNA. As shown in Fig. 3, the presence of sperm DNA completely eliminated the interaction of IRs probe with BosR. In contrast, the mobility of both *cisI* and *cisII* probes was restrained by BosR, a result that clearly indicates that both *cisI* and *cisII* are able to effectively bind BosR.

Next, the *ospAB* regulatory sequences were compared for BosR binding affinity. The IRs probe was first added as a competitor. As shown in Fig. 3, although IRs DNA was added at a concentration of 10-fold greater than *cisI* or *cisII*, it did not significantly reduce the interaction of BosR with either *cisI* or *cisII*, reiterating the results showing that IRs do not bind with BosR. When *cisI* and *cisII* probes were added as a competitor, *cisI* was unable to significantly reduce the interaction of *cisII* with BosR, but the addition of *cisII* essentially eliminated the interaction of *cisI* with bosR, allowing for the conclusion that *cisII* more effectively binds BosR than *cisI*.

Our previous study showed no effect of the presence or absence of Zn^2+^ on the DNA-binding activity of BosR [16]. Nevertheless, the influence of Zn^2+^ on the binding of BosR to both *cisI* and *cisII* was investigated. At concentrations below 10 µM, Zn^2+^ had no effect on binding. When Zn^2+^ was added to 100 µM, however, the binding of BosR to either *cisI* or *cisII* was significantly inhibited (date not shown).

### Excessive BosR expression causes cell death in *B. burgdorferi*

To specifically regulate BosR expression, pIBM-*bosR_in_* was constructed as illustrated in Fig. 1A. Within the construct, BosR expression was under the control of an inducible promoter. Production of BosR should not occur from the construct in the absence of the inducer IPTG, although the native *bosR* gene may produce BosR as normal. The construct pIBM-*bosR_in_* was easily introduced into Δ*rpoS*. This mutant was used because any RpoS involvement in OspA downregulation was readily ruled out in this way. It was also electroporated into 13A, the parental clone of Δ*rpoS*, as a control. In a single transformation experiment with Δ*rpoS*, five transformants were obtained. Plasmid analyses led to the identification of one clone, namely, Δ*rpoS/bosR_in_*, which lost cp9, lp5, lp21, lp25 and lp56 as Δ*rpoS*, in addition to lp28-1. Transformation of 13A led to the selection of seven transformants, one of which, namely 13A/*bosRin*, lost cp9, lp5, lp21, lp25, lp56 and lp28-1 and was chosen for further studies. There has been no evidence that any of these lost plasmids affects gene regulation although both lp25 and lp28-1 are critical for murine infection [26]. However, there is a possibility that additional complexity of Osp-BosR regulation exists in wild-type spirochetes.

**Figure 4:**
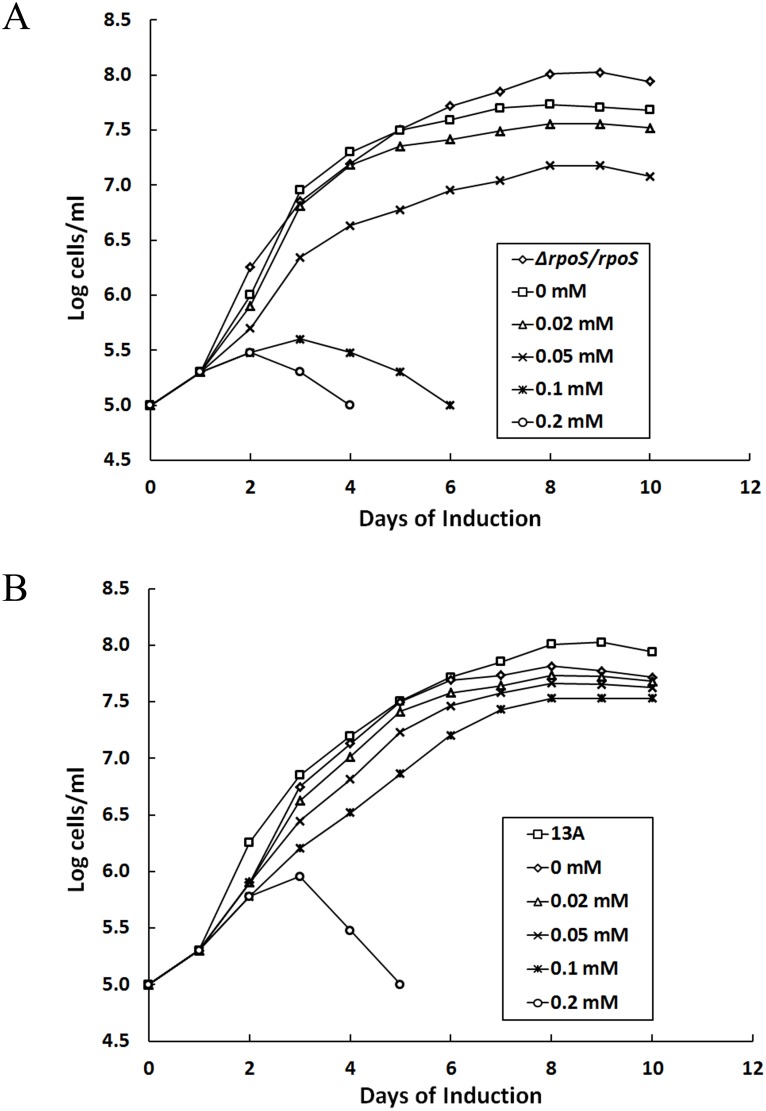
Excessive BosR expression causes cell death. (A) A total of 15 1.3-ml aliquots of Δ*rpoS/bosR_in_* spirochetes at a density of 10^5^ cells/ml were prepared and supplemented with IPTG at final concentrations of 0, 0.02, 0.05, 0.10, and 0.20 mM. Each concentration was in triplicate. The 15 aliquots were incubated at 33°C and cell numbers were counted daily for 10 days. Mean counts presented here were calculated from the triplicates of each treatment. The Δ*rpoS/rpoS* spirochetes were used as a control. (B) The same experimental design was used to examine the 13A/*bosR’* spirochetes when the parental clone 13A was used as a control.

Our previous study reported that increasing RpoS expression causes cell death [27]. To investigate whether high BosR expression is toxic, both Δ*rpoS/bosR_in_* and 13A/*bosR_in_* were grown to early log phase (10^7^ cells/ml) in BSK-H medium and diluted to 10^5^ cells/ml before IPTG was added to final concentrations ranging from 0 to 0.2 mM. When IPTG reached as low as 0.05 mM, Δ*rpoS/bosR_in_* growth was affected (Fig. 4A). When the concentration increased to 0.1 mM, growth was essentially arrested within a couple of days after induction and all spirochetes eventually died during the subsequent week. Although at lower concentrations IPTG did not affect early growth, its presence reduced the stationary cell density.

At 0.1 mM, the inducer showed little effect on growth of 13A/*bosR_in_*, indicating that this strain was less sensitive than Δ*rpoS/bosR_in_* to induction with IPTG (Fig. 4B). Even at 0.2 mM, the 13A/*bosR_in_* spirochetes continued to grow for a couple of days before beginning to die; induced spirochetes became uncountable within a week.

### Inducing BosR expression leads to dramatic downregulation of OspA

**Figure 5:**
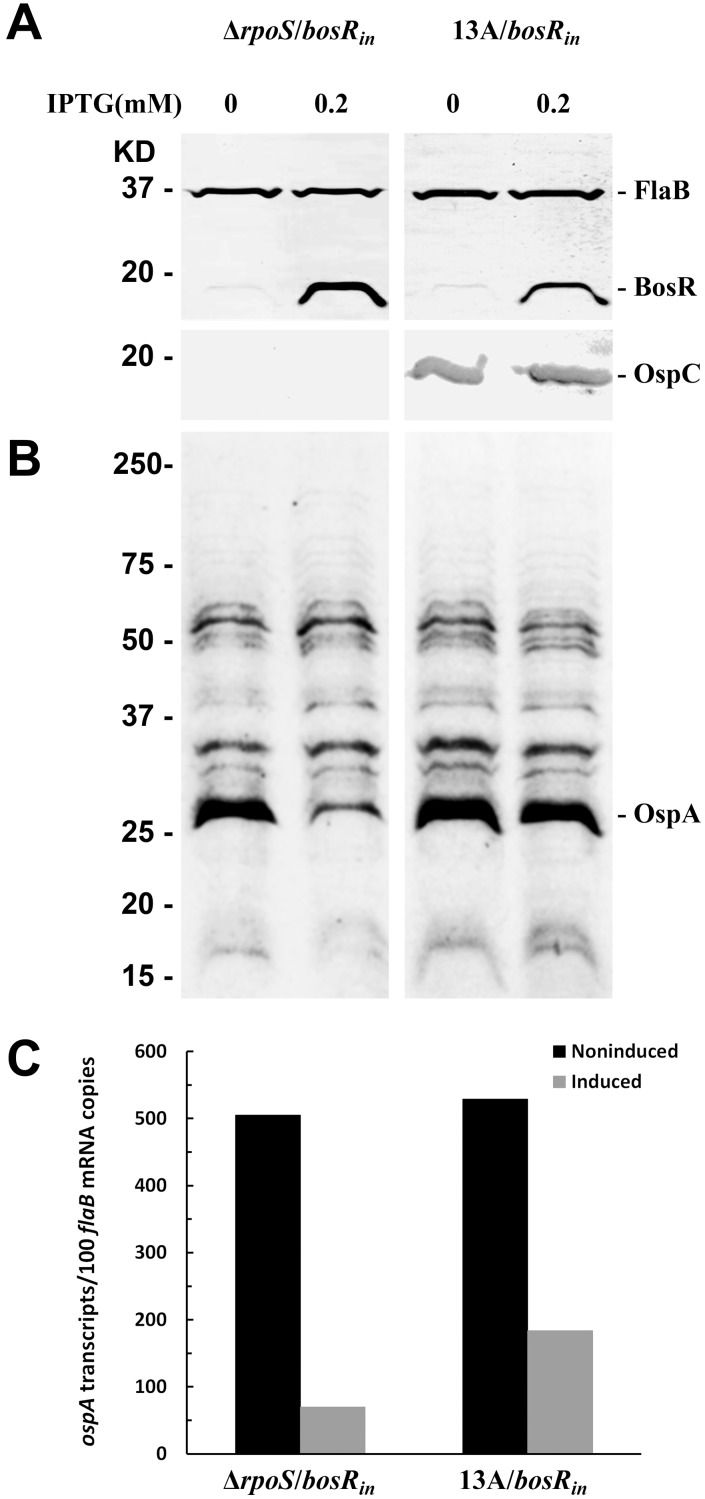
Inducing BosR expression leads to dramatic downregulation of OspA. (A&B) Both Δ*rpoS/bosR_in_* and 13A/*bosR_in_* spirochetes were grown to a density of 10^7^ cells/ml and then supplemented with IPTG at final concentrations of 0 or 0.2 mM. Induction was allowed to proceed for 3 days. Treated bacteria were harvested and analyzed either by immunoblotting probed with a mixture of FlaB mAb and mouse BosR antibodies (A, upper panel) or with OspC mAb alone (A, lower panel), or Coomassie staining (B). (C) Total RNA was extracted from induced and noninduced spirochetes 24 hours after initial treatment, converted to cDNA and analyzed by qRT-PCR.

As shown above, excessive BosR expression caused a lethal consequence to *B. burgdorferi*, therefore it is important to use appropriate concentrations of the inducer to treat the bacteria. At 0.2 mM, IPTG significantly inhibited both Δ*rpoS/bosR_in_* and 13A/*bosR_in_* growth shortly after induction and killed them within a week. This concentration was chosen for the investigation of how induction of BosR influenced OspA expression. The Δ*rpoS/bosR_in_* and 13A/*bosR_in_* spirochetes were grown to 10^7^ cells/ml before supplementing with IPTG at 0.2 mM. Induction was allowed to proceed for three days, during which time the spirochete density reached approximately 10^8^ cells/ml, reflecting a three-fold increase. As shown in Fig. 5, as BosR was induced, OspA expression dramatically reduced in Δ*rpoS/bosR_in_*, but had a lesser effect in 13A/*bosR_in_*. However, the reduction in the detected protein amount may primarily depend on the dilution effect of cell division as OspA may be stable in live cells. Therefore, a reduction in the total OspA amount may not be greater than three-fold even if the transcriptional process could be fully stopped immediately upon induction. Moreover, the abundant *ospA* mRNA accumulated before induction would continue to be translated into protein until it was degraded.

Total RNA was also prepared and analyzed. As shown in Fig. 5C, induced BosR expression led to a reduction in *ospA* transcription greater than 86% in Δ*rpoS/bosR_in_* and 65% in 13A/*bosR_in_*. Unlike protein, mRNA is less stable and its level would be better reflected in the magnitude of *ospA* gene downregulation resulting from increased BosR expression.

Although induction led to a dramatic increase in BosR production, OspC expression did not significantly increase (Fig. 5A), probably because RpoS was also regulated by other regulators, such as RpoN, small RNAs and BadR [13], [28]–[30]. An unbalanced increase in BosR expression might not significantly increase RpoS production if the other regulators were not actively involved.

### Increasing BosR expression completely shuts off OspA production when constitutive expression of other Osps simultaneously occurs

Although increasing BosR expression, even to a toxic level, leads to dramatic downregulation of OspA, it does not abolish it. To completely downregulate OspA with increased BosR, we designed an experiment based on our hypothesis that the Osp layer homeostasis of *B. burgdorferi* overwrites the normal regulatory programs. In other words, as OspAB are the most dominantly expressed Osps in cultured spirochetes, *B. burgdorferi* cannot completely downregulated them without dramatically increasing expression of other Osps. Based on the hypothesis, two additional constructs, pBBE22-*bosR’* and pBBE22-*C’B’A’*-*bosR’*, were created as shown in Fig. 1. Within the former, the fused *bosR* gene was designed to express driven by the *flaB* promoter, while in the latter, in addition to a fused *bosR* gene, three *osp* genes, *ospC*, *dbpA* and *dbpB*, were all engineered to transcribe under the control of a fused *flaB* promoter.

**Figure 6:**
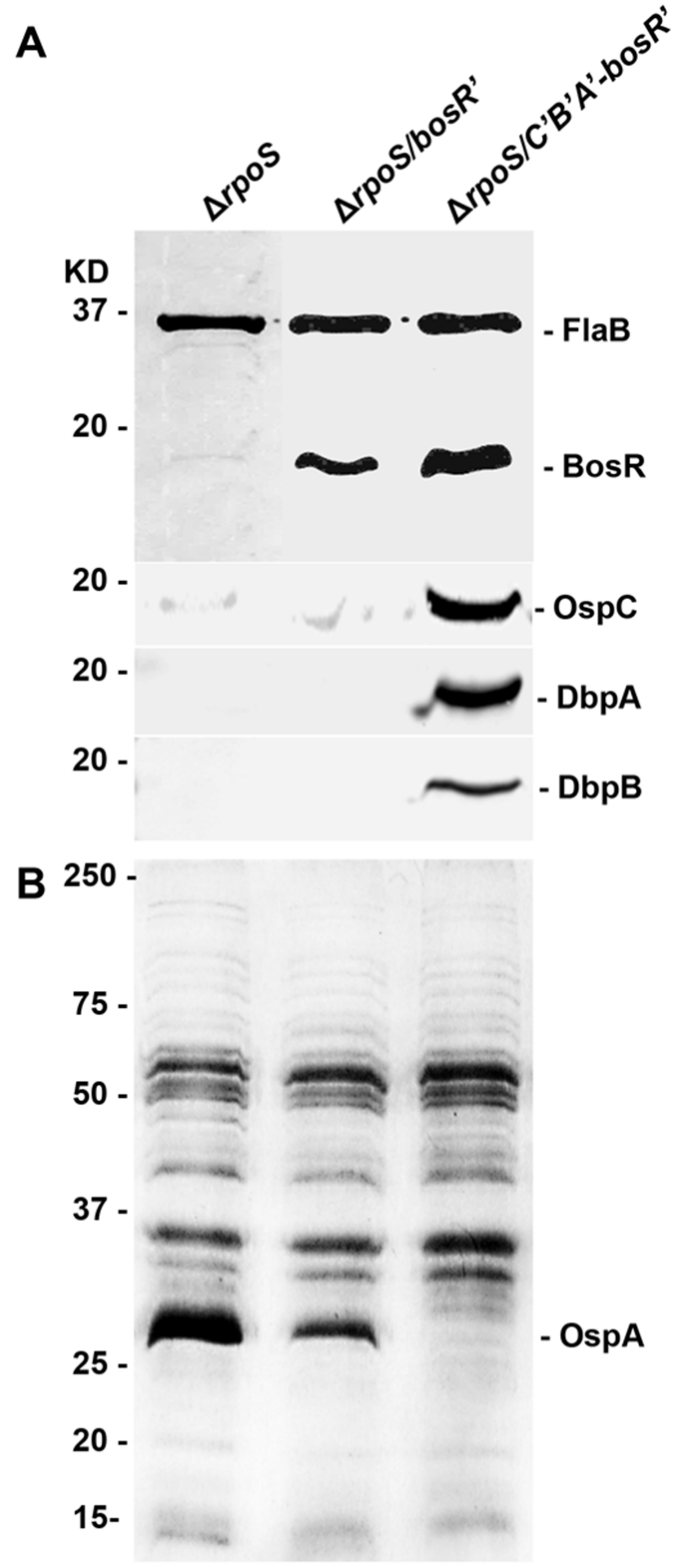
Increasing BosR expression shuts off OspA production only when other Osps, OspC, DbpA and DbpB, are constitutively expressed. The Δ*rpoS*, Δ*rpoS/bosR’* and Δ*rpoS/C’B’A’-bosR’* were grown to late log phase (10^8^ cells/ml), harvested and analyzed either by immunoblotting probed with FlaB, BosR, OspC, DbpA and DbpB antibodies (A), or Coomassie staining (B).

After these constructs were introduced into Δ*rpoS*, five and eight transformants were obtained, respectively. Plasmid analyses led to the identification of two clones, namely, Δ*rpoS/bosR’* and Δ*rpoS/C’B’A’-bosR’*, which lost cp9, lp5, lp21, lp25 and lp56 as Δ*rpoS*. In addition, both clones also lost lp28-1. As shown in Fig. 6, a high level of BosR expression driven by the fused *flaB* promoter greatly downregulated OspA, but was unable to completely shut off expression, consistent with the result obtained with the inducible promoter shown in Fig. 5. In the presence of constitutive expression of OspC, DbpA and DbpB, increased BosR production led to complete shutoff of OspA production.

## Discussion

OspA is highly immunogenic and would induce a strong immune response even if expressed at a very low level during mammalian infection [31], [32]. Even though OspA antibodies may not effectively kill the spirochetes with low OspA expression in mammalian tissues, once acquired by the tick vector, the pathogen is extremely sensitive to the specific antibodies [5], [33]. In order to maintain its infectious life cycle, *B. burgdorferi* must abundantly produce OspA during this stage in the tick vector and downregulate to below the immune system detectable level during infection of the mammalian host. The current study revealed OspA downregulation can be achieved through binding BosR to the regulatory elements of the *ospAB* operon. Clearly, BosR functions as a repressor and the two regulatory elements, *cisI* and *cisII*, serve as operators of the *ospAB* operon.

Our previous study identified both *cisI* and *cisII* as the regulatory sequences of the *ospAB* operon and showed that the presence of both was required for maximum downregulation during murine infection [15]. The study also showed that the two elements do not contribute equally to *ospA* downregulation as *cisII* exhibits a four-fold greater reduction in *ospA* transcription when compared to *cisI* [15]. These previous findings appear consistent with those of the current study, indicating that both elements bind BosR with *cisII* having more binding affinity than *cisI*. This double-operator system with use of a single common repressor may ensure full shutoff of the *ospAB* operon and, thus providing the pathogen with a secure mechanism to completely conceal the highly immunogenic antigens during mammalian infection.

BosR is required for expression of RpoS, which in turn activates *ospC*, *dbpBA* and many other genes encoding Osp proteins [17], [19]. The regulator is not expressed in early growth phases, but is dramatically upregulated in the late log phase. No significant OspA downregulation has been observed in cultured spirochetes, despite BosR reaching its highest level in the stationary phase. Even in the tick’s midgut, no more than 50% of the spirochete population downregulate OspA during any period of the transmission bloodmeal [4]. Most of the remaining spirochetes highly express OspC, an indication that BosR is actively expressed, but they do not show OspA downregulation. Given these previous observations, the process of inducing OspA downregulation should not be oversimplified just based on the fact *B. burgdorferi* readily shuts off OspA either during mammalian infection or being grown in the host-adapted conditions [31], [34]. As a matter of fact, to induce OspA downregulation, BosR expression was increased to a level that nearly kills *B. burgdorferi*. Under this *in vitro* condition, OspA expression was significantly downregulated but was not shut off.

When cycling between the two distinct hosts, *B. burgdorferi* must adapt bacterial survival strategies to extremely different environments. Without a doubt, spirochetal adaptation must dramatically change its gene expression. Under normal conditions, complete OspA downregulation occurs only during mammalian infection, in which OspC and other RpoS-dependent Osps are dramatically upregulated. The mammalian host apparently provides an extreme environment as these specific alternation signals, which may be unachievable under any *in vitro* conditions. While either excessive RpoS or BosR expression is lethal to *B. burgdorferi in vitro*, these high levels of expression may be essential for the pathogen to achieve downregulation of the *ospAB* operon and to greatly upregulate RpoS-dependent virulence factors, and ultimately allow *B. burgdorferi* to survive better in the mammalian host. To achieve OspA downregulation in *B. burgdorferi* grown *in vitro*, a high level of BosR expression in combination with simultaneous expression of OspC and other Osps may be required.

One feature of *B. burgdorferi* is to coat itself with lipoproteins. A decade ago, we hypothesized that the pathogen must maintain its Osp expression level in order to keep it viable [35]. In other words, *B. burgdorferi* must upregulate other Osps to compensate for the loss resulting from downregulation of some Osps. Our previous study designed based on the hypothesis successfully revealed dramatic upregulation of VlsE with OspC expression being shut off by *B. burgdorferi* reacting to an amounting specific humoral response [35]. Another study based on the same hypothesis successfully restored OspC-deficient spirochetes with infectivity by increasing expression of an Osp, such as OspA, VlsE, DbpA or ErpA [36]. Based on the same hypothesis, we designed experiments and modified the Δ*rpoS* spirochete to simultaneously expression BosR and three major Osps to successfully achieve full shutoff of OspA in *B. burgdorferi* grown under *in vitro* conditions.

As a key regulator, BosR, like RpoS, must be strictly regulated. As the current study demonstrated, BosR causes cell death when expressed at a very high level as RpoS does [27]. The cell death caused by excessive RpoS expression may be simply attributed to *σ* factor competition, but there is no simple explanation for BosR-related death. While the biological significance of induced cell death remains to be addressed, the possibility that it is to control cell populations less diverse in the same environment should be considered. For instance, the death strategy would select out subpopulations with a phenotype that highly expresses RpoS- or BosR-dependent genes when in the tick vector. However, cell death resulting from increased RpoS or BosR expression is observed only *in vitro*. Given that *B. burgdorferi* cycles between the extremely different environments encountered in the tick vector and a mammal, *in vitro* growth conditions may only constitute an abnormal environment. As emphasized above, it is possible that a high level of BosR and RpoS expression may provide an essential strategy for the pathogen to survive. Especially during infection of the mammalian host, *B. burgdorferi* may have to highly produce BosR to achieve shutoff of OspAB expression, as well as dramatic upregulation of RpoS-dependent virulence genes, in order to adapt to the extreme environment.

An RpoS-deficient background was used initially to rule out any involvement of RpoS in OspA downregulation, as a previous study suggested the alternative *σ* factor may be involved in the regulation [14]. The current study clearly showed that there is no direct involvement of RpoS in OspA downregulation. BosR positively affects expression of many Osps, including OspC, DbpA and DbpB, through activation of RpoS expression [17], [18]. We did expect that induced BosR expression would more effectively cause downregulation of OspA in 13A/*bosR_in_* than Δ*rpoS/bosR_in_* as BosR also increases RpoS expression, which, in turn, increases OspC, DbpA and DbpB expression and increased production of these Osps would compensate for the loss of OspAB. However, the current study showed that induced BosR more effectively causes OspA downregulation in Δ*rpoS/bosR_in_* than 13A/*bosR_in_*, suggesting that gene regulation in *B. burgdorferi* is more complicated than previously thought. Our immunoblotting result showed BosR expression significantly stronger in the RpoS-deficient background than in 13A/*bosR_in_*, suggesting that the presence of RpoS may produce a negative feedback on BosR expression. Such a feedback may include multiple regulators, which are either directly or indirectly regulated by RpoS.

As a critical regulator, BosR binds the *rpoS* promoter region and activates its transcription with an involvement of other regulators, such as RpoN. The current study demonstrated that via binding of the two regulatory sequences of the *ospAB* operon, BosR shuts off gene expression. Elucidating the function of this regulator, which operates in two opposite ways, remains to be addressed. Our previous study showed the presence of *cisI* and *cisII* enhanced *ospA* transcription in spirochetes grown *in vitro*, albeit the effect was very minor, suggesting that BosR may be involved in upregulation of the gene, suggestive of another regulatory function of BosR when it is expressed at a relatively low level [15].

Taken together with our previous study showing that the presence of both *cisI* and *cisII* is required for maximum *ospA* downregulation in the murine host [15], the current study clearly demonstrates BosR functioning as a repressor of the *ospAB* operon by binding both regulatory elements and shutting off OspA expression. In combination with a series of studies by others showing that BosR positively regulates many Osps via upregulation of RpoS [17], [19], the current study provides sufficient evidence allowing for the conclusion that BosR, in general, functions as a coordinator by indirectly upregulating RpoS-dependent Osps, such as OspC, DbpA and DbpB, and directly repressing expression of OspAB.
